# Dutch-Cantonese Bilinguals Show Segmental Processing during Sinitic Language Production

**DOI:** 10.3389/fpsyg.2017.01133

**Published:** 2017-07-07

**Authors:** Kalinka Timmer, Yiya Chen

**Affiliations:** ^1^Center for Brain and Cognition, Pompeu Fabra UniversityBarcelona, Spain; ^2^Leiden University Centre for Linguistics, Leiden UniversityLeiden, Netherlands; ^3^Leiden Institute for Brain and Cognition, Leiden UniversityLeiden, Netherlands

**Keywords:** speech production, bilingualism, segmental processing, syllabic processing, EEG/ERP

## Abstract

This study addressed the debate on the primacy of syllable vs. segment (i.e., phoneme) as a functional unit of phonological encoding in syllabic languages by investigating both behavioral and neural responses of Dutch-Cantonese (DC) bilinguals in a color-object picture naming task. Specifically, we investigated whether DC bilinguals exhibit the phonemic processing strategy, evident in monolingual Dutch speakers, during planning of their Cantonese speech production. Participants named the color of colored line-drawings in Cantonese faster when color and object matched in the first segment than when they were mismatched (e.g., 藍駱駝, /**l**aam4/ /**l**ok3to4/, “blue camel;” 紅饑駝, /**h**ung4/ /**l**ok3to4/, “red camel”). This is in contrast to previous studies in Sinitic languages that did not reveal such phoneme-only facilitation. Phonemic overlap also modulated the event-related potentials (ERPs) in the 125–175, 200–300, and 300–400 ms time windows, suggesting earlier ERP modulations than in previous studies with monolingual Sinitic speakers or unbalanced Sinitic-Germanic bilinguals. Conjointly, our results suggest that, while the syllable may be considered the primary unit of phonological encoding in Sinitic languages, the phoneme can serve as the primary unit of phonological encoding, both behaviorally and neurally, for DC bilinguals. The presence/absence of a segment onset effect in Sinitic languages may be related to the proficiency in the Germanic language of bilinguals.

## Introduction

Many models of speech production recognize, despite their differences, that there are three main stages of speech planning: the formation of the concept to be conveyed; the retrieval of phonological representations; and the articulation of the planned speech (Stemberger, 1985; Dell, 1988; Roelofs, 1997; Levelt et al., 1999). One of the debates in the last two decades is what constitutes as the primary planning unit in cross-linguistic online speech production during the phonological encoding stage. The present study aims to shed further light on the issue by investigating whether the primary processing unit is universal and to what extent it is determined by one's language background. We explore this question with bilinguals speaking two different types of languages (Germanic and Sinitic). Further, with the use of ERPs, we investigate the neural activation pattern of the phoneme underlying the presence or absence of behavioral effects which existing debates on primary processing unit have mainly relied upon. In the following, we first introduce the existing literature on Germanic languages which suggested the phoneme to be universally activated in all languages, which is followed by a description of behavioral research in Sinitic languages that challenges this universality view and suggests that the planning unit is language-specific. We then discuss (1) how understanding processing mechanisms of bilinguals with different language backgrounds may shed light on the issue, and (2) how ERPs would give us more insight into the activation pattern of segmental information.

In West-Germanic languages, the phoneme has been found to serve as the primary unit of phonological planning. Initial evidence comes from speech error analyses indicating a large portion of phoneme-sized insertion, deletion, and substitution errors in English (Shattuck-Hufnagel and Klatt, 1979). Further evidence comes from experimental paradigms which reported faster responses in conditions with phoneme onset overlap than no overlap conditions. For example, in the picture-word interference paradigm participants named pictures faster when they were superimposed by a word that matched with the first phoneme of the picture (e.g., Schriefers et al., 1990; Meyer and Schriefers, 1991; Damian and Martin, 1999; Starreveld, 2000). Also in other production tasks, like the implicit priming paradigm (Meyer, 1991; Roelofs, 1999, 2003) and the color-object picture naming task (Damian and Dumay, 2007, 2009), overlap of the onset phoneme facilitated speech latencies. Phoneme facilitation was also found during reading aloud tasks that used priming (e.g., Forster and Davis, 1991; Kinoshita, 2000; Schiller, 2004, 2008; Timmer and Schiller, 2012; for a review see Timmer and Schiller, 2014). Taken together, the earlier consensus in the literature was that the phoneme serves as the primary unit of phonological encoding during on-line speech production. The prevalence of evidence for initial phoneme encoding in native speakers of West-Germanic languages, such as Dutch and English, has led to the assumption that the initial phoneme activation during phonological encoding is a universal feature. Dominant models of speech production include this assumption (Roelofs, 1997; Levelt et al., 1999).

Several studies investigating Sinitic languages have challenged the universality of phoneme as a functional unit of planning. Natural slips of the tongue in Mandarin Chinese rarely cause phoneme-sized errors (Chen, 2000). The same experimental paradigms used in Western languages discussed above did not find evidence of phoneme onset facilitation in varieties of Sinitic languages. For example, no facilitation was found in the implicit priming paradigm (Chen et al., 2002 for Mandarin, Chen et al., 2002; O'Seaghdha et al., 2010; Cantonese: Wong et al., 2012), the picture-word interference paradigm (Wong and Chen, 2008 for Cantonese), the primed word naming paradigm (Chen and Li, 2011 for Mandarin), the primed picture-naming task (Yu et al., 2014 for Mandarin), or the color-object picture naming task (Qu et al., 2012 for Mandarin). More specifically, in the latter paradigm, participants were asked to name the color and object of a picture drawing. No facilitation was present when the first phoneme of the color and object matched. O'Seaghdha et al. (2010) therefore states that Germanic and Sinitic languages differ in the proximate unit for initial phonological processing. Phonological encoding during lexical retrieval in languages such as Mandarin, a Sinitic language, seems to begin with syllable retrieval, which constitutes as the initial functional unit of phonological encoding, which subsequently is followed by retrieval of individual phonemes within the syllable frame for articulation. In languages such as Japanese, this initially retrieved syllabic unit is called a mora (e.g., Kureta et al., 2006; Verdonschot et al., 2011). In contrast to Mandarin and Japanese, in languages such as English, a Germanic language, the phoneme is retrieved as the primary functional unit.

The dissociation between syllable and phoneme facilitation also seems to be supported by the different scripts and phonotactics of Sinitic vs. West-Germanic languages. In Germanic languages, the phoneme can serve as a more efficient functional unit of processing. Given that Sinitic languages are syllabic and re-syllabification is far less common than in Germanic languages, it is probably more feasible to access the entire syllable in Sinitic languages, as an efficient functional unit during phonological encoding. Thus, the primary functional unit of phonological encoding has been suggested to be language specific (O'Seaghdha et al., 2010).

It is important to note that speech perception studies have revealed an interestingly dynamic role that a processing unit can play in spoken word recognition (Cutler et al., 2001). Studies on bilinguals have shown that whether the processing unit in their dominant language may be used during the processing of the other language is contingent upon the language-specific characteristics of the languages involved (Cutler et al., 1989, 1992). For example, English-French bilinguals were observed to use their English processing strategy when listening to French. However, French-English bilinguals used different strategies depending on the language spoken because the French strategy would have been inefficient during English processing.

Within the speech production literature, the debate between the language-universal vs. language-specific functional unit of phonological encoding can also benefit from further research on bilingual speakers, especially bilingual speakers of languages which have been proposed to have different functional units of phonological encoding. In a masked priming experiment, native Mandarin speakers who were highly proficient in English, showed phoneme onset priming in their production of English (Verdonschot et al., 2013). For highly proficient Japanese-English bilinguals the same pattern was revealed, showing sensitivity to the phoneme onset in English (Ida et al., 2015; Nakayama et al., 2016). This suggests that native speakers of a syllabic/moraic language can employ different processing units depending on the language used (i.e., a phoneme-sized unit for phonological encoding in English). Interestingly, while the Japanese-English bilinguals adhered to their native mora-based processing strategy in Japanese (Ida et al., 2015), Mandarin-English bilinguals showed phoneme onset priming even in their production of Chinese (although contingent upon syllable structure sharing between the prime and target) instead of only syllable priming (Verdonschot et al., 2013). One question that arises is what might have modulated the adaptive behavior of bilingual speakers. The question may be adjudicated with more insights from neurophysiological studies.

ERP studies with bilinguals from the same population as discussed above, namely Mandarin-English bilinguals with high-level proficiency in English, reported significant neural responses to segmental repetition in Mandarin Chinese, despite the lack of segmental priming effect in response time (Qu et al., 2012; Yu et al., 2014). Specifically, Qu and colleagues found that initial segment repetition in a color-object picture naming task elicited more positive ERPs in the posterior regions during the 200–300 ms time window and more negative ERPs in the anterior regions during the 300–400 ms time window after picture onset, relative to no-repetition trials. Yu and colleagues reported that overlapping phonemes in a picture-naming priming task in both the initial and non-initial position evoked more positive ERPs in the 180–300 ms interval throughout the whole scalp as well as more negative ERPs in the mid-anterior regions in the 350–450 ms interval. These ERP components are claimed to be in agreement with previous ERP studies on overt speech production, as shown in results of meta analyses of phonological encoding and internal monitoring by Indefrey and Levelt (2004) and Indefrey (2011).

fMRI evidence with Mandarin speakers also argued for the distinctive neural representations of phonemes and syllables (Yu et al., 2015; see also findings in Peeva et al., 2010 on distinct activation patterns for phoneme and syllable in French). For phoneme activation both studies show activation of the pallidum and putamen. Yu shows additional activation of the STg region, which seems specifically activated for Chinese languages (Fu et al., 2002). While we note that the interpretation of cognitive processes based on neurophysiological observations has to be taken with caution (Munding et al., 2016) the above mentioned studies seem to suggest that there is a potentially more important role of the segment in speech encoding for Sinitic languages than previously suggested, which raises the question of the mechanism that explains the lack/presence of phoneme repetition priming effect in the behavioral response time data.

Qu et al. (2012) proposed an account that maintains segment as a functional unit of planning even in Sinitic languages which involves overriding phonological activation by a monitoring process (hereafter the Monitoring Account). This was endorsed by Yu et al. (2014). The effect during the 180–300 ms time window found by Yu et al. (2014) could be related to the P2, which reflects lexical access (e.g., Indefrey and Levelt, 2004; Hirschfeld et al., 2008; Costa et al., 2009; Strijkers et al., 2010; Aristei et al., 2011). The P2 is, for example, manipulated by cognate status with greater positivity for non-cognates than cognates (Strijkers et al., 2010 and descriptively reported in Christoffels et al., 2007). Due to the clear phonological overlap for cognates, both representations are strongly co-activated. A feedback loop sends phonological activation back to phonologically linked lexical representations, but this does not happen for unrelated words or non-cognates (e.g., Dell, 1986; Levelt et al., 1998; Costa et al., 2005). Taken together, smaller P2 seems to reflect easier retrieval of phonologically related words in general. The second component found by Qu et al. (2012) and Yu et al. (2014) could reflect self-monitoring, a process that cancels out the facilitations created in the P2 which leads to the lack of behavioral segmental onset facilitation.

There are also alternative accounts explaining these observations. Roelofs (2015) proposed an account that recognizes the universal role of segmental planning and explains away the null effect of segment repetition priming in languages like Mandarin by assuming that segmental activation was hidden by the parallel selection of the other segments of the first syllable during the planning of the actual response (hereafter the Concurrent Retrieval Account). Another account, from a very different perspective, is to attribute the lack of RT evidence to the lack of intentional orientation toward segments in Mandarin Chinese and attribute the observed phoneme repetition ERP effects as an index of phonological connectivity rather than functional engagement of segments in preparation for production. This approach was proposed by O'Seaghdha et al. (2013) (hereafter the Connectivity Account), but was refuted by Qu et al. (2012) as neuro-physiologically infeasible. It is important to note that the participants in Qu et al. (2012) and Yu et al. (2014) are university students, who learned Chinese mainly via the Latin alphabet-based Pinyin system. Nowadays, they also mainly use the Pinyin input method on digital systems to type the logographic Chinese characters, which potentially could have boosted their sensitivity to phonemic representations of Chinese characters.

More empirical data, especially those that tap both into the behavioral and neural patterns of phonological encoding, are crucially needed to resolve the debates. Therefore, the current study aims to extend the body of literature by examining Dutch-Cantonese bilinguals. The segment-retrieval hypothesis has been consistently supported in studies with Dutch-speaking individuals. On the contrary, Cantonese speakers have consistently shown a lack of initial phoneme onset priming, despite evidence that sub-syllabic units, such as consonant-vowel (CV) and rhyme, may serve as possible processing units (Wong and Chen, 2008, 2009; Wong et al., 2012). Specifically, we were interested in whether balanced Dutch-Cantonese bilinguals would demonstrate initial segment-based serial planning during speech production in their syllabic language (Cantonese) in the behavioral data. Furthermore, we were interested in how such an influence manifests itself in the ERP neural response patterns. In doing so, we aimed to bring in new data that may shed light on the existing debates concerning the universality of segment as a functional unit of speech encoding, and the possible mechanism that explains the mismatch of phoneme repetition priming effect between the ERP and behavioral responses.

**Figure 1:**
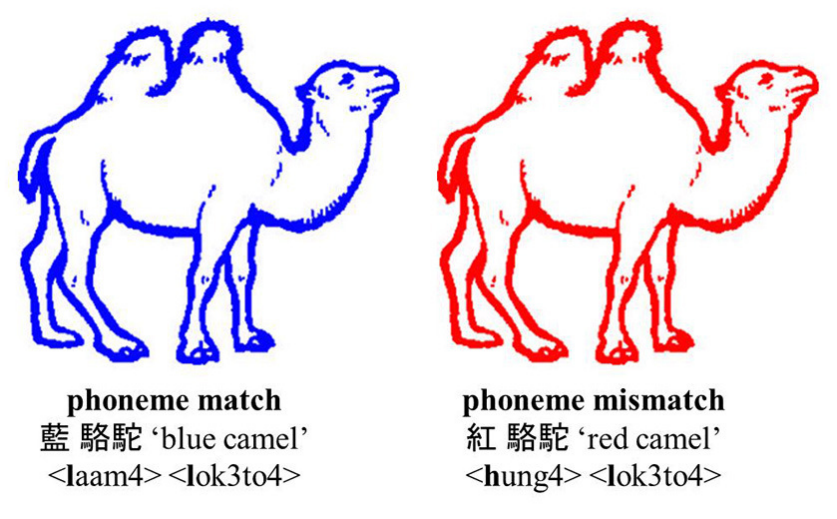
Example of a colored object in the two experimental conditions: phoneme match and phoneme mismatch. The Cantonese character, English translation, and *jyutping* (i.e., Romanized system for Cantonese). The picture has been adjusted from Severens et al. (2005).

We used a naming task in which participants were asked to name the color of colored line drawings of objects in Cantonese^1^. The relationship between color and object was manipulated in such a way that the onset of the color and object was phonologically related or -unrelated (see Figure 1). We expected to see not only ERP differences (i.e., evidence of segment as a functional unit of phonological processing), but also behavioral facilitation (i.e., evidence of segment being the proximate unit of processing) as has been found in Dutch. Furthermore, we were interested in how ERP evidence on initial segment priming may differ from or confirm findings in Qu et al. (2012) and Yu et al. (2014).

## Methods

### Participants

The data from 18 bilingual speakers of Dutch and Cantonese Chinese (four females; average age = 23.9; *SD* = 3.37) were used in the analyses. Out of the 23 subjects who participated in the experiment the data from five participants were rejected due to technical problems (*n* = 3), extremely slow responses (above 2 *SD*s of the group mean; *n* = 1), or being left handed (*n* = 1). All participants have normal or corrected-to-normal vision. None of them were color-blind or had a history of neurological impairments or language disorders. The Dutch-Contonese bilinguals were from the Netherlands and the experiment was conducted in the Netherlands as well. All participants were proficient in both Cantonese and Dutch. Cantonese was learned at home and at Cantonese Saturday school. For 14 participants, Cantonese was the mother tongue for both parents and for four participants one of the parents spoke Cantonese as a mother tongue. On average participants went to Cantonese school for 9.5 years (*SD* = 2.92). Dutch was learned at school as all participants were born in the Netherlands or moved there before the age of school. They attended Dutch schools and followed the same curriculum as other Dutch children. These characteristics of their language experience essentially render the participants as balanced bilinguals, as they are proficient early speakers of both Cantonese and Dutch. See Table 1 for an overview of their language proficiency as rated by a self-rated proficiency questionnaire adapted from Christoffels et al. (2007).

**Table 1:** Mean answers (and standard deviations) to the self-rating proficiency questionnaire (range: 0–10 or 100%).

	**Dutch**	**Cantonese**
Age starting to learn Dutch/Cantonese	3.5 (1.87)	1.5 (2.79)
Active skills Dutch/Cantonese	9.1 (1.13)	8.3 (1.49)
Passive skills Dutch/Cantonese	8.9 (1.48)	7.9 (0.96)
% speaking Cantonese during a day		40.9 (20.86)
% reading Cantonese during a day		13.5 (12.84)
% listening Cantonese during a day		43.2 (23.45)

### Materials

Forty-eight black-and-white line drawings of objects were selected from various databases including Snodgrass Vanderwart (Snodgrass and Vanderwart, 1980), Els Severens (Severens et al., 2005), and Alario picture set (Alario and Ferrand, 1999). The color paired with the line drawing was not its canonical color (e.g., tomato was not paired with a red line). Each line drawing was presented in two of eight colors (red, orange, yellow, green, blue, purple, gray, and black) to create two conditions: (1) the first phoneme of the color and line drawing match in Cantonese (e.g., 藍駱駝, /**l**aam4/ /**l**ok3to4/, “blue camel;” phoneme-match), and (2) the first phoneme mismatches in Cantonese (e.g., 紅駱駝, /**h**ung4/ /**l**ok3to4/, “red camel;” phoneme-mismatch; see Figure 1). The color names in Cantonese were monosyllabic, and all picture names were disyllabic. Tonal mismatch between the color name and first syllable of the picture name was attempted. Due to the small number of depictable pictures and the phonological matching between color and object in Cantonese but not Dutch, there are some pairs which had tonal overlap, which, nevertheless are comparably distributed in the match condition (14 pairs) and the mismatch condition (10 pairs). Further, there are seven pairs that overlap in the second phomeme between the color and the noun (i.e., five match pairs and two mismatch pairs). Note that when the pairs with phoneme match on the second phoneme are the removed the results are the same (see results). All the stimuli are represented in the **Appendix**.

For the practice phase, eight pictures, which were not part of the 48 experimental pictures, were presented in one of the eight colors. The first phoneme of the color name always mismatched that of the line drawing.

### Design and procedure

The study was approved by the ethics review board at Leiden University. Participants first signed an informed consent form and filled out a self-rated language proficiency questionnaire. They were tested individually in a quiet room seated ~90 cm from the computer screen. The experiment was controlled by the software package E-Prime 2. Speech production onset was measured though an integrated voice-key (microphone).

The experiment consisted of three parts: (1) learning phase (48 trials), (2) practice phase (48 trials), and (3) experimental phase (96 trials). Each phase was preceded by eight practice trials. During the learning phase, eight color patches, followed by 56 drawings of objects, were presented with their corresponding Cantonese characters. Both the color patches and drawings were presented in random order. The participant made sure they knew the names and the experimenter pressed a button to continue to the next color patch/drawing. During the practice phase, the same color patches and drawings were presented one by one in random order. Participants were asked to name them as fast and accurately as possible. During the experimental phase, the drawings were presented one by one in one of eight colors. Participants only named the color of color-object drawings as first used by Navarrete and Costa (2005).

Each trial in the experimental phase consisted of a fixation-cross (400–700 ms), followed by a color-drawing that disappeared once the participant initiated a verbal response or after a time-out of 3,000 ms, after which a blank screen was presented for 2,000 ms. All pictures were 10 × 10 cm and centered on the screen.

During the test phase each drawing was presented twice, once in the color where the phoneme matches the drawing and once in the mismatching color. All drawings appeared in each of two created blocks. In each block, half of the pictures were presented in the phoneme-match and the other half in the phoneme-mismatch condition. Blocks and trials were randomized over participants.

### Apparatus and data acquisition

The electroencephalogram (EEG) signals were sampled at 512 Hz and continuously recorded using 32 Ag/AgCl electrodes distributed according to the extended International 10–20 system. Two electrodes of the flat type (above and below the left eye) recorded the eye-blinks. Another two electrodes (external canthi of each eye) recorded horizontal eye-movements. The EEG signal was re-referenced to the mastoids (left and right; baseline).

### Data analysis

For the EEG analysis, epochs of 600 ms with an additional 200 ms pre-stimulus baseline were created. The EEG signal was filtered with a high-pass filter of 0.01 Hz/24 dB and a low-pass filter of 40 Hz/24 dB. Ocular artifacts were corrected using the Gratton et al. (1983) algorithm. Non-ocular artifacts were removed based on the following criteria: trials with amplitudes below −200 μV, above +200 μV, or made a voltage step of 100 μV within 200 ms. The ERP grand averages were time-locked to the onset of the target word and calculated separately for each of the two conditions over all participants.

**Figure 2:**
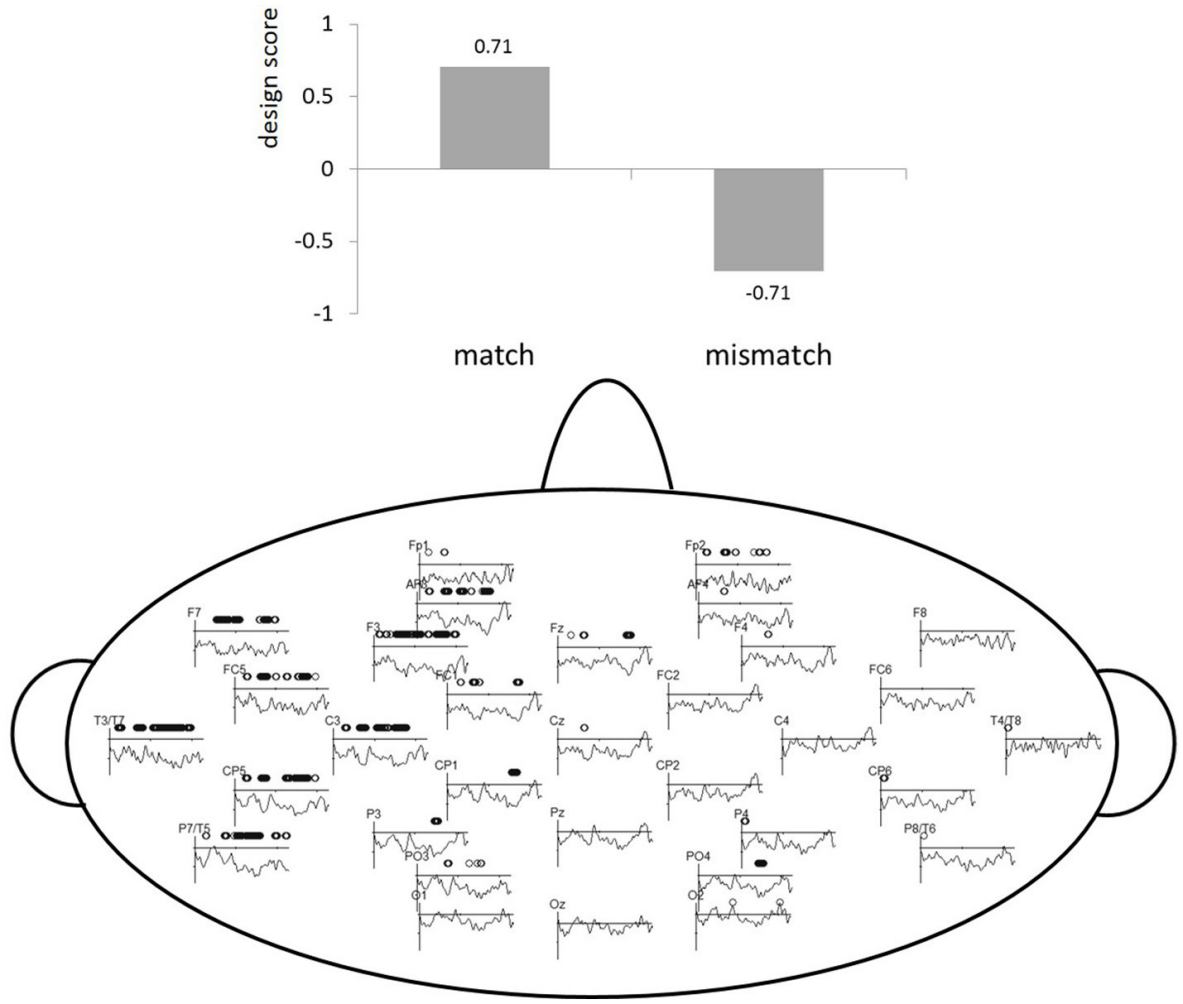
PLS results. The **top** part of the figure represents the design scores for the LV (y-axis). The **bottom** part of the figure indicates a PLS electrode saliency map showing the reliability of LV for the match vs. mismatch comparison. The x-axis represents time in milliseconds (0–450) and the y-axis represents electrode salience (i.e., reliability of the LV).

To avoid any *a priori* bias with respect to choosing time windows and localization for ERP analyses, which allows for vast number of comparisons, a multivariate statistical tool called partial least squares (PLS) was used (McIntosh et al., 1996; Lobaugh et al., 2001; Krishnan et al., 2011). All ERP data is submitted to PLS by importing microvolts for every ms from stimulus presentation to 450 ms for each electrode. This is done for all participants and conditions. Singular value decomposition (SVD) identifies a set of latent variables (LVs), that correspond to the strongest ERP patterns in the data based on our experimental contrast/conditions (match vs. mismatch). An LV explains how much of the covariance was explained by our experimental contrast. To visualize the LVs, the salience is computed and represented as design scores and salience maps. Design scores code the effects of the LV between −1 and 1 (see top part of Figure 2) and salience plots show where this effect is present spatiotemporally (bottom part od Figure 2). In the salience maps, the dots show for which electrodes and temporal windows the LV is explaining the covariance. Thus, the relation between the experimental design contrasts (represented by the LV) and the spatiotemporal pattern of ERP amplitude changes is represented by the electrode saliences in Figure 2. The estimate of obtaining a singular value by chance (similar to a *p*-value) was computed by 1,000 permutations. The reliability of electrode saliences at each time point was assessed by 200 bootstrap re-samplings, which applies random sampling with replacement. For examples of how PLS can be applied to EEG data, see Lobaugh et al., 2001; Hay et al., 2002; Düzel et al., 2003; Itier et al., 2004; Grundy and Shedden, 2014 and for an example for speech production ERP data see, Christoffels et al. (2016). For more detailed explanation of applications and formulas see McIntosh and Lobaugh (2004). In short, PLS analyses allowed us to narrow the time windows and locations of experimental effects in order to perform conventional ERP statistics.

**Figure 3:**
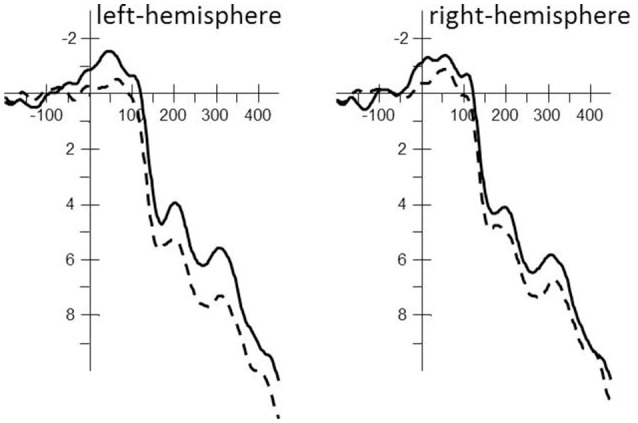
Averaged stimulus-locked ERP waveforms for the phoneme match (solid line; e.g., 藍駱駝 /**l**aam4/ /**l**ok3to4/, “blue camel”) and phoneme mismatch condition (dashed line; e.g., 紅駱駝 /**h**ung4/ /**l**ok3to4/, “red camel”) for each hemisphere including all electrodes used in the statistical analysis. A 20 Hz filter was applied for the clarity of the waveforms.

The independent factor Phoneme condition (match vs. mismatch) was first examined with whole-brain PLS analysis within the interval. The LV suggests that the phoneme match and mismatch trials were processed differently (see Figure 2) and accounted for 100% of the variance, as this design only has one latent variable, *p* < 0.05. The electrode saliences, reflecting confidence intervals for salience across time points and electrodes, revealed that this effect was most reliable within the 125–175, 200–300, and the 300–400 ms time windows throughout the left temporal hemisphere (electrodes F7, FC5, T7, C3, CP5, and P7). Based on correspondence with PLS, we chose an analogous location in the right hemisphere for subsequent componential analysis. These time-windows were analyzed by a classic statistical ERP analyses with Phoneme condition (match vs. mismatch) and Hemisphere (left: F7, FC5, T7, C3, CP5, P7 vs. right: F8, FC6, T8, C4, CP6, P8) as independent variables and ERPs measured in μV as the dependent variable. The Greenhouse–Geisser correction was applied to all repeated measures to correct for possible violations of sphericity. Note that based on visual inspection of Figure 3, the 0–100 ms time window suggests possible differences, but neither PLS nor classic ERP analysis revealed significant effects of this time window.

## Results

### Behavioral data

Naming latencies shorter than 200 ms and longer than 1,000 ms, voice-key errors, and incorrect responses (4.4% of the data) were discarded from the analysis, leaving a total of 95.6% of the trials in the analysis.

The independent factor Phoneme condition (match vs. mismatch) with the dependent variable, RTs, were submitted to a repeated-measures ANOVA, which revealed that the colors of phonologically related color-object pairs (e.g., 藍駱駝, /**l**aam4/ /**l**ok3to4/, “blue camel;” 784 ms; SE = 30.14) were named 20 ms faster compared to phonologically unrelated pairs [e.g., 紅駱駝, /**h**ung4/ /**l**ok3to4/, “red camel;” 804 ms; SE = 32.27; *F*_(1, 17)_ = 7.12, *p* = 0.016]^2^.

### ERP data

Trials that included incorrect responses or electrophysiological artifacts were removed from the analysis. For the phoneme match condition 11.34% of the data was removed, leaving 88.66% in the analysis. For the phoneme mismatch condition 10.42% of the data was removed, leaving 89.58% in the analysis.

### 125–175 ms time window

The repeated-measures ANOVA revealed a main effect of Phoneme condition [*F*_(1, 17)_ = 5.35, *MSe* = 22.24, *p* < 0.05] that interacted with Localization [*F*_(1, 17)_ = 6.64, *MSe* = 1.08, *p* < 0.05]. The phoneme mismatch condition revealed greater positive amplitudes than the phoneme match condition throughout the left-hemisphere [*F*_(1, 17)_ = 8.84, *MSe* = 10.44, *p* < 0.01; phoneme match: μV = 3.10; SE = 0.71 vs. phoneme mismatch: μV = 4.41; SE = 0.71] but not the right hemisphere [*F*_(1, 17)_ = 2.63, *MSe* = 12.88, *ns*; phoneme match: μV = 2.98; SE = 0.78 vs. phoneme mismatch: μV = 3.78; SE = 0.83; see Figure 3].

### 200–300 ms time window

The repeated-measures ANOVA revealed a main effect of Phoneme condition [*F*_(1, 17)_ = 4.82, *MSe* = 30.14, *p* < 0.05] that interacted with Localization [*F*_(1, 17)_ = 5.15, *MSe* = 1.67, *p* < 0.05]. The phoneme match condition revealed greater negative amplitudes than the phoneme mismatch condition throughout the left-hemisphere [*F*_(1, 17)_ = 8.62, *MSe* = 13.03, *p* < 0.01; phoneme match: μV = 5.37; SE = 0.97 vs. phoneme mismatch: μV = 6.81; SE = 0.95] but not the right hemisphere [*F*_(1, 17)_ = 2.22, *MSe* = 18.78, *ns*; phoneme match: μV = 5.67; SE = 1.14 vs. phoneme mismatch: μV = 6.55; SE = 1.17; see Figure 3].

### 300–400 ms time window

The repeated-measures ANOVA revealed a main effect of Phoneme condition [*F*_(1, 17)_ = 4.45, *MSe* = 28.36, *p* = 0.05] that interacted with Localization [*F*_(1, 17)_ = 9.73, *MSe* = 1.49, *p* < 0.01]. The phoneme match condition revealed greater negative amplitudes than the phoneme mismatch condition throughout the left-hemisphere [*F*_(1, 17)_ = 9.63, *MSe* = 11.74, *p* < 0.01; phoneme match: μV = 7.11; SE = 1.02 vs. phoneme mismatch: μV = 8.55; SE = 1.01] but not the right hemisphere [*F*_(1, 17)_ = 1.52, *MSe* = 18.11, *ns*; phoneme match: μV = 7.19; SE = 1.08 vs. phoneme mismatch: μV = 7.91; SE = 1.17; see Figure 3].

## Discussion

The present study investigated segment-based serial planning mechanism during syllabic language (Cantonese) speech planning by Dutch-Cantonese bilinguals. Our results show a behavioral facilitation for phoneme onset sharing in Cantonese, a syllabic language, for Dutch-Cantonese bilinguals. This is in contrast to behavioral results with monolingual Cantonese speakers where segmental onset facilitation is normally absent (Wong and Chen, 2008; Wong et al., 2012). Our finding is probably due to the native proficiency of our participants in Dutch, a segment-based language in which phoneme onset effects are found (Schriefers et al., 1990; Meyer, 1991; Meyer and Schriefers, 1991). Their experience with Dutch facilitates segment production in Cantonese. Thus, our bilinguals seemed to be able to utilize their segment-oriented production strategy used in Germanic languages and apply it to their syllabic language, Cantonese. This is in line with a previous study showing that Mandarin-English bilinguals with a good proficiency in English can demonstrate initial segmental facilitation during reading aloud in Mandarin under specific conditions (e.g., same tonal pattern). However, Ida et al. (2015) were not able to replicate this effect with proficient Japanese-English bilinguals in Japanese. Thus, there is mixed evidence that the unit size of English as a second language can exhibit influence on that of L1 speech processing. The present study is the first to show phoneme onset facilitation in Cantonese speech production for native speakers of a segment-based language. This may suggest that the primary processing unit in a specific language is dependent on the speakers's general language background. Within spoken word recognition bilinguals have also shown to use the unit of lexical access from their dominant language in their second language if it is an efficient processing strategy for that language (Cutler et al., 1989, 1992).

With regard to the ERP neural responses, our bilingual DC speakers showed earlier activation of ERP components compared to previous studies with bilingual Mandarin-English speakers. The bilinguals in the present study are native speakers of a Germanic language, while in previous studies second language learners of a Germanic language were investigated (Qu et al., 2012; Yu et al., 2014). Thus, differences in proficiency may explain a divergence of activation in the ERPs. Specifically, both Yu et al. (2014) and Qu et al. (2012) reported the first significant differences between phoneme match and mismatch conditions from ~190 ms after picture presentation. In the present study, however, phonological processing was first revealed during the 125–175 ms time window. Phoneme mismatch trials induced greater positive amplitudes compared to the phoneme match condition. This component could reflect a P2 with easier retrieval of phonologically related words in general. While a meta-analysis of neurophysiological studies has demonstrated a pattern of processing steps of among others lexical access and phonological encoding during speech production (Indefrey, 2011), it has to be noted that the link between cognitive operations and neurophysiological observations has to be taken with caution (Munding et al., 2016).

While Qu et al. (2012) only analyzed components after ~190 ms in Figure 1C of their article, visual inspection of the figure suggests that an earlier P2 is present around ~125 ms after picture onset with a slightly greater positivity for the phoneme mismatch than match condition in the anterior regions. This is in line with the results in the current study, although it needs to be confirmed with further analysis of this P2 component. The trend in Qu et al. (2012) and the results of the present study suggest a primary role for segmental planning early on during lexical access for highly proficient Dutch speakers in the syllabic language of Cantonese. A potential though not robust effect of segment overlap on P2 in Qu's study (visually also smaller than that in the present study) could be attributed to the experience of her participants with Pinyin as well as English which are both segment-based and could have introduced certain degrees of enhanced sensitivity to the phoneme.

Yu et al. (2014), however, revealed a later onset of the P2 component, around 180 ms after picture onset, with the opposite pattern of conditions (i.e., greater positivity for the phoneme match than mismatch condition). This pattern is probably due to the fact that they used a different paradigm than color only naming in the present study, or color-object naming, used in Qu et al.'s (2012) study. During their experiment, disyllabic pictures were named one after the other, while the phonological *onset* relationship was manipulated between consecutive pictures. Therefore, the temporal distance between the prime and target picture presentation includes the inter-trial-interval (ITI; 600 ms) and fixation cross (500 ms). The presentation of the phonological prime at least 1,100 ms before the target created a different situation from the previously mentioned studies where the prime (color) and target (object) are presented at the same time. Therefore, it is plausible that the early segmental effect in the present study is introduced during lexical access (P2), as the bilinguals adopted the segmental encoding which they acquired by speaking a segment-oriented language (i.e., Dutch).

The P2 is followed by a negative component between 200 and 300 ms with greater negativities for the phoneme match compared to the phoneme mismatch condition. This component, commonly named N2 (or descriptively named N3 by Strijkers et al., 2010), reflects phonological encoding during language production. During this phonological encoding stage speakers continuously monitor whether their phonological output is correct. Multiple lexical or phonological representations are available during this process. Therefore, conflict can arise from co-activation of phonological representation of color and object. In order to correctly produce an utterance the conflict must be resolved. This conflict could possibly be resolved through inhibition. This is similar to effects found with cognates vs. non-cognates (Christoffels et al., 2007; Strijkers et al., 2010; and descriptively reported in Verhoef et al., 2009) and during a word interference paradigm (Hoshino and Thierry, 2011). When producing a lexical item other lexical items or phonological representations are activated as well and this has to be resolved. Despite the conflicts, all of the above mentioned paradigms show behavioral facilitation for phonological matching conditions. The N2 effect has also been related to general response inhibition (e.g., Pfefferbaum et al., 1985; Jodo and Kayama, 1992; Thorpe et al., 1996) as well as to response conflict monitoring (e.g., Nieuwenhuis et al., 2003; Donkers and Van Boxtel, 2004). Thus, our results can be taken as evidence that the phonological overlap between color and object causes inhibition or response conflict monitoring during the N2 component, in line with the existing literature.

The last time window, 300–400 ms, coincides with that of Qu et al. (2012) and Yu et al. (2014) who also investigated segment sized phonological activation during speech production in syllabic languages. In their monitoring account they suggest this component could reflect self-monitoring. A higher cognitive load is suggested to be present for the phoneme-related condition, because they can cause speech errors, and is suggested to cancel out the small segment facilitation effect in the behavioral data where no segmental-priming effect is observed (Qu et al., 2012; Yu et al., 2014).

To summarize, the present study revealed behavioral segmental onset facilitation as well as early facilitation of repeated segment-sized phonemes during lexical access, reflected in the P2 component. This is followed by two negative components where overlapping phonology causes inhibition and additional self-monitoring. This raises the question of how onset overlap can be named faster than mismatched cases while introducing more response conflict during the later ERP components, especially given the findings in research with manual responses that have demonstrated delayed responses after conflict (e.g., Tillman and Wiens, 2011). There seem to be two important differences between manual and speech response. First, speech responses are not as diverse as manual responses. During manual responses there are usually two completely different responses (e.g., left vs. right hand response), while during speech production, response options involve similar vocal tracts and articulators. Second, the motor responses of speech develop differently over time than manual responses. Facilitative relationship of the two phonologically related representations can be observed earlier in time. Only later the two representations start diverging (Acheson et al., 2012). This could explain why the later competition (demonstrated in the ERPs) in our data does not have the same impact on speech onset latencies as on the speed of manual responses in the case for non-linguistic tasks on the speed of manual responses (e.g., Tillman and Wiens, 2011).

Further, the P2 present in the current experiment also showed a visual trend in Qu et al.'s (2012) study but with a seemingly smaller differences of P2 compared to the current study. It could therefore be that early activation of the segment was not strong enough for the speakers of Mandarin in their study to show behavioral facilitation, probably due to the lower level of their proficiency in a segment-oriented language (i.e., English). In contrast, our bilingual DC speakers grew up in the Netherlands and so, their Dutch has reached native/near native proficiency. Thus, while our study lends support to the proposal in Qu et al. (2012) that segment can serve as a functional unit of planning even in Sinitic languages such as Cantonese, we believe that the lack of phonological activation (as indexed by the reaction time) in their study is not likely explained by the Monitoring Account. With regard to the debate between the Concurrent Retrieval Account (Roelofs, 2015) and the Connectivity Account (O'Seaghdha et al., 2013), neither would have predicted the behavioral phoneme onset facilitation effect in our bilingual speakers. Further experimental evidence is needed to test the extent of their feasibility.

To conclude, the behavioral data, together with the P2, provided evidence that Dutch-Cantonese bilinguals used the segment as the primary unit of planning during Cantonese speech planning. The results of the present study are compared to previous studies. However, a direct comparison with the present study was not possible due to task differences and participants' different language experiences. For example, we reported earlier ERP evidence during the lexical accessing stage for segmental processing than previous research with unbalanced bilinguals, who are less proficient in an alphabetic language (Qu et al., 2012; Yu et al., 2014). Our results have been interpreted and discussed within the existing literature and theories. In future research, a more direct comparison is preferable. Specifically, our proposal that proficiency of bilingual speakers in one language can modulate the adaptive behavior of processing unit in another certainly needs corroborative evidence from studies that tap directly into the effect of proficiency level on phonological encoding, preferably with planned comparisons of processing patterns in different languages within the same bilingual populations. In this way, further insights into the cross-linguistic functional unit(s) of phonological planning can be obtained. Future endeavors should also explore whether a timing difference can be shown between segmental and syllabic activation within and between Germanic and syllabic languages. To conclude, the language background of bilinguals can have a significant impact on the primary processing unit during speech production in a specific language.

## Ethics statement

The experiments reported in our manuscript were conducted within the ethical regulations of the University of Leiden. Prior to participation all participants were informed about the procedure and signed an informed consent form.

## Author contributions

KT and YC: substantial contributions to the conception and design of the manuscript: interpretation of data for the manuscript; drafting the manuscript and revising it critically; Agreement to be accountable for all aspects of the work in ensuring that questions related to the accuracy or integrity of any part of the work are appropriately investigated and resolved.

### Conflict of interest statement

The authors declare that the research was conducted in the absence of any commercial or financial relationships that could be construed as a potential conflict of interest.

## Footnotes

^1^In the present study participants only named the color of the color-object pairs, as in Navarrete and Costa (2005), instead of both the color and object (e.g., Qu et al., 2012). There may be processing differences between color only or color-object naming tasks. However, both show behavioral phonological facilitation.

^2^Note that when the pairs that have phoneme overlap on the second phoneme are removed we see the same results. Phonologically related color-object pairs (783 ms; SE = 29.34) were named 19 ms faster compared to phonologically unrelated pairs [802 ms; SE = 31.45; *F*_(1, 17)_ = 6.07, *p* = 0.025].

## Appendix

**Table:**

**Phoneme-match**	**Phoneme-mismatch**
黑口琴 /haak1/ /hau2 kam4/ “black harmonica”	藍口琴 /laam4/ /hau2 kam4/ “blue harmonica”
黑熊貓 /haak1/ /hung4 maau1/ “black panda”	藍熊貓 /laam4/ /hung4 maau1/ “blue panda”
黑香腸 /haak1/ /hoeng1 coeng2/ “black sausage”	灰香腸 /fui1/ /hoeng1 coeng2/ “gray sausage”
黑校服 /haak1/ /haau6 fuk6/ “black school uniform”	黃校服 /wong4/ /haau6 fuk6/ “yellow school uniform”
黑海星 /haak1/ /hoi2 sing1/ “black starfish”	綠海星 /luk6/ /hoi2 sing1/ “green starfish”
黑河馬 /haak1/ /ho4 maa5/ “black hippopotamus”	灰河馬 /fui1/ /ho4 maa5/ “gray hippopotamus”
藍駱駝 /laam4/ /lok6 to4/ “blue camel”	紅駱駝 /hung4/ /lok6 to4/ “red camel”
藍禮物 /laam4/ /lai5 mat6/ “blue present”	黑禮物 /haak1/ /lai5 mat6/ “black present”
藍樓梯 /laam4/ /lau4 tai1/ “blue stairs”	黑樓梯 /haak1/ /lau4 tai1/ “black stairs”
藍辣椒 /laam4/ /laat6 ziu1/ “blue chilli pepper”	灰辣椒 /fui1/ /laat6 ziu1/ “gray chilli pepper”
藍輪椅 /laam4/ /leon4 ji2/ “blue wheelchair”	黃輪椅 /wong4/ /leon4 ji2/ “yellow wheelchair”
藍拉鏈 /laam4/ /laai1 lin2/ “blue zipper”	黃拉鏈 /wong4/ /laai1 lin2/ “yellow zipper”
灰飛機 /fui1/ /fei1 gei1/ “gray airplane”	橙飛機 /caang2/ /fei1 gei1/ “orange airplane”
灰斧頭 /fui1/ /fu2 tau4^*^2/ “gray ax”	紫斧頭 /zi2/ /fu2 tau4^*^2/ “purple ax”
灰風扇 /fui1/ /fung1 sin3/ “gray fan”	橙風扇 /caang2/ /fung1 sin3/ “orange fan”
灰火柴 /fui1/ /fo2 caai4/ “gray match”	綠火柴 /luk6/ /fo2 caai4/ “green match”
灰花生 /fui1/ /faa1 sang1/ “gray peanut”	綠花生 /luk6/ /faa1 sang1/ “green peanut”
灰番茄 /fui1/ /faan1 ke2/ “gray tomato”	藍番茄 /laam4/ /faan1 ke2/ “blue tomato”
綠蠟燭 /luk6/ /laap6 zuk1/ “green candle”	橙蠟燭 /caang2/ /laap6 zuk1/ “orange candle”
綠榴槤 /luk6/ /lau4 lin4/ “green durian”	橙榴槤 /caang2/ /lau4 lin4/ “orange durian”
綠老鼠 /luk6/ /lou5 syu2/ “green mouse”	黃老鼠 /wong4/ /lou5 syu2/ “yellow mouse”
綠螺絲 /luk6/ /lo4 si1/ “green screw”	紅螺絲 /hung4/ /lo4 si1/ “red screw”
綠領帶 /luk6/ /leng5 daai2/ “green tie”	黑領帶 /haak1/ /leng5 daai2/ “black tie”
綠喇叭 /luk6/ /laa3 baa1/ “green trumpet”	紅喇叭 /hung4/ /laa3 baa1/ “red trumpet”
橙擦膠 /caang2/ /caat3 gaau1/ “orange eraser”	綠擦膠 /luk6/ /caat3 gaau1/ “green eraser”
橙彩虹 /caang2/ /coi2 hung4/ “orange rainbow”	灰彩虹 /haak1/ /coi2 hung4/ “black rainbow”
橙鞦韆 /caang2/ /cau1 cin1/ “orange swing”	黑鞦韆 /fui1/ /cau1 cin1/ “gray swing”
橙茶杯 /caang2/ /caa4 bui1/ “orange teacup”	綠茶杯 /laam4/ /caa4 bui1/ “blue teacup”
橙廁所 /caang2/ /ci3 so2/ “orange toilet”	綠廁所 /luk6/ /ci3 so2/ “green toilet”
橙窗口 /caang2/ /coeng1 hau2/ “orange window”	黑窗口 /hung4/ /coeng1 hau2/ “red window”
紫炸彈 /zi2/ /zaa3 daan6^*^2/ “purple bomb”	黃炸彈 /wong4/ /zaa3 daan6^*^2/ “yellow bomb”
紫磚頭 /zi2/ /zyun1 tau4/ “purple brick”	灰磚頭 /fui1/ /zyun1 tau4/ “gray brick”
紫鑽石 /zi2/ /zyun3 sek6/ “purple diamond”	紅鑽石 /haak1/ /zyun3 sek6/ “black diamond”
紫嘴唇 /zi2/ /zeoi2 seon4/ “purple lips”	黃嘴唇 /wong4/ /zeoi2 seon4/ “yellow lips”
紫枕頭 /zi2/ /zam2 tau4/ “purple pillow”	黑枕頭 /haak1/ /zam2 tau4/ “black pillow”
紫酒杯 /zi2/ /zau2 bui1/ “purple wine cup”	藍酒杯 /laam4/ /zau2 bui1/ “blue wine cup”
紅氣球 /hung4/ /hei3 kau4/ “red balloon”	灰氣球 /fui1/ /hei3 kau4/ “gray balloon”
紅香蕉 /hung4/ /hoeng1 ziu1/ “red banana”	紫香蕉 /zi2/ /hoeng1 ziu1/ “purple banana”
紅汽車 /hung4/ /hei3 ce1/ “red car”	藍汽車 /laam4/ /hei3 ce1/ “blue car”
紅海豚 /hung4/ /hoi2 tyun4/ “red dolphin”	橙海豚 /caang2/ /hoi2 tyun4/ “orange dolphin”
紅荷花 /hung4/ /ho4 faa1/ “red lotus”	紫荷花 /zi2/ /ho4 faa1/ “purple lotus”
紅汽水 /hung4/ /hei3 seoi2/ “red soft drink”	紫汽水 /zi2/ /hei3 seoi2/ “purple soft drink”
黃碗碟 /wong4/ /wun2 dip6/ “yellow bowls and dishes”	橙碗碟 /caang2/ /wun2 dip6/ “orange bowls and dishes”
黃蝴蝶 /wong4/ /wu4 dip6/6^*^2/ “yellow butterfly”	藍蝴蝶 /luk6/ /wu4 dip6/6^*^2/ “green butterfly”
黃狐狸 /wong4/ /wu4 lei4^*^2/ “yellow fox”	紅狐狸 /hung4/ /wu4 lei4^*^2/ “red fox”
黃蝸牛 /wong4/ /wo1 ngau4/ “yellow snail”	紫蝸牛 /zi2/ /wo1 ngau4/ “purple snail”
黃泳池 /wong4/ /wing6 ci4/ “yellow swimming pool”	紫泳池 /zi2/ /wing6 ci4/ “purple swimming pool”
黃烏龜 /wong4/ /wu1 gwai1/ “yellow tortoise”	紅烏龜 /hung4/ /wu1 gwai1/ “red tortoise”
